# The Dilemma in the Management of Patients with Heart Failure with Reduced Ejection Fraction, Sinus Rhythm and Left Ventricular Spontaneous Echo Contrast: A Narrative Review

**DOI:** 10.2174/011573403X363285250519064030

**Published:** 2025-05-27

**Authors:** Hedieh Alimi, Ali Tajik

**Affiliations:** 1 Faculty of Medicine, Cardiovascular Research Center, Mashhad University of Medical Sciences, Mashhad, Iran;; 2 Faculty of Medicine, Student Research Committee, Mashhad University of Medical Sciences, Mashhad, Iran

**Keywords:** Left ventricular, heart failure, spontaneous echo contrast, thromboembolism, anticoagulation, sinus rhythm

## Abstract

Heart failure (HF) is a complex clinical syndrome that arises from structural or functional impairment of ventricular filling or ejection of blood, resulting in previous characteristic symptoms of fatigue, dyspnea, and fluid retention. Among the complications of heart failure is the development of spontaneous echo contrast (SEC), characterized by a smoke-resembling appearance on echocardiograms, which indicates blood stasis in heart chambers. Despite being identified as an echocardiographic marker in the left atrium that correlates with thrombus formation and causes thromboembolic events, the clinical importance of left ventricular spontaneous echo contrast (LV-SEC) and the appropriate management for patients with this condition remain uncertain due to insufficient data. Anticoagulant therapy is generally recommended for patients with established left ventricular thrombus (LVT). However, for patients with heart failure with reduced ejection fraction (HFrEF) and sinus rhythm (SR), as a result of a decrease in thromboembolic events over time, it is typically not recommended. The main challenge lies in assessing the thromboembolic risk and determining appropriate management in patients with HFrEF, sinus rhythm (SR), and left ventricular spontaneous echo contrast (LV-SEC), compared to those with left ventricular thrombus (LVT) and those with HFrEF and SR without LV-SEC. The aim of this paper is to review the guidelines and trials on clinical characteristics, outcomes, and management of patients with LV-SEC and compare the suggested management with the established management for LVT and HF patients with sinus rhythm without LV-SEC.

## 1 INTRODUCTION

Heart failure (HF) is a complex clinical syndrome characterized by structural or functional impairment of ventricular filling or ejection, manifesting in symptoms such as fatigue, dyspnea, and fluid retention [1]. HF is characterized by symptoms and/or signs resulting from a structural and/or functional cardiac abnormality, which could be systolic or diastolic and corroborated by elevated natriuretic peptide levels and/or objective evidence of pulmonary or systemic congestion. Systolic heart failure consists of three categories, namely HF with reduced ejection fraction (HFrEF), mildly reduced EF (HFmrEF), and preserved EF (HFpEF), according to the EF ranges <_40%, 41–49%, and >_50%, respectively [2, 3].

**Fig. (1):**
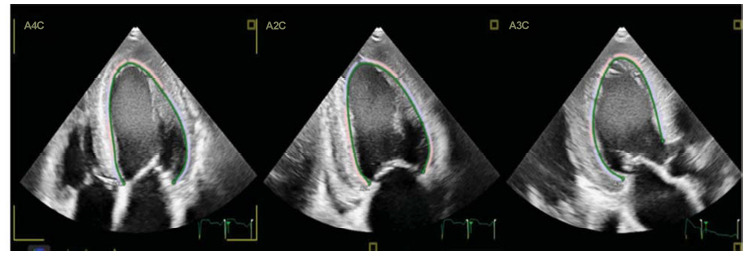
Left ventricular spontaneous echo contrast (LV-SEC) in a patient with severe LV systolic dysfunction. Smoke-like density appears on echocardiography in apical four (A4C), two (A2C), and three (A3C) chamber views, indicating stasis of blood in the left ventricle with low left ventricular strain.

A complication of HF is spontaneous echo contrast (SEC), a smoke-like density on echocardiograms indicating blood stasis in the heart chambers (Fig. **1**) [4]. This echocardiographic marker has been found to be a precursor of thrombus formation and causes thromboembolic events in patients with heart failure, even with normal sinus rhythm [5]. A growing body of evidence has indicated that there is a strong association between left atrial SEC (LA-SEC) and an elevated risk of thromboembolism [6, 7]. However, there are insufficient data on the clinical characteristics and significance of LV-SEC. Risk stratification in patients with left ventricular SEC (LV-SEC) can be employed to discern specific patient subgroups that may benefit from prompt intervention. This approach could potentially alleviate the social and economic burden related to thromboembolic events. This review aimed to summarize the published evidence regarding the use of anticoagulation in patients with HFrEF, sinus rhythm (SR), and LV-SEC.

## 2 MATERIALS AND METHODS

In order to identify relevant articles, we accomplished a thorough search in databases like Web of Science, Google Scholar, Scopus, PubMed, and Central. The keywords were “left ventricular”, “heart failure”, “spontaneous echo contrast”, “thromboembolism”, “anticoagulation”, and “sinus rhythm”. English-language articles were selected based on title and abstract screening, with no restrictions on publication status or start date. The literature search was conducted up to March 31, 2024. The duplicate articles were removed using Endnote 21. Consequently, the pertinent articles were reviewed.

### 2.1 Pathogenesis, Risk Factors, and Prevalence of SEC

Due to the widespread utilization of echocardiography for the diagnosis of heart diseases, a noteworthy observation has been made regarding the video density within the cardiac chambers. Occasionally, a swirling, smoke-like density of varying intensity might be observed. This phenomenon, known as spontaneous echo contrast, results from ultrasonic backscatter caused by erythrocyte aggregates [8, 9]. The formation of these aggregates is a result of noncovalent binding between erythrocytes and plasma proteins, mainly fibrinogen [10], which has a positive correlation with low flow conditions while displaying an inverse relationship with shear rate [11, 12]. Additionally, in 2001, Zotz *et al*. [13] aimed to ascertain the origin of SEC through direct investigation of left atrial blood. The study demonstrated a notable elevation in left atrial platelet and leukocyte activation in patients with SEC, as shown by increased surface antigen expression on these cells. Furthermore, an increased amount of platelet-leukocyte aggregation in the left atrium among individuals with SEC was noted due to heightened activation status. The mechanisms responsible for the creation of SEC continue to be a subject of contention. Blood cell aggregates are thought to be responsible for SEC, although there is debate about whether red cell or platelet aggregates are the principal contributors to this phenomenon.

Various grading systems have been employed to evaluate SEC, primarily focusing on the left atrium. For example, 0 indicates no smoke, 1+, mild smoke visible in some portion of the chamber, and 2+, dense smoke that appears throughout the entire chamber [14]. An alternative system describes grade 0 as no SEC, grade 1 as mild, grade 2 as moderate, and grade 3 as severe [15]. Another scoring system for LA-SEC includes five grades: 0, defined as non or absence of echogenicity, 1 defined as mild or minimal echogenicity, 2 defined as mild to moderate, which has a more dense swirling pattern than 1 but has a similar distribution, 3 defined as a moderate or dense swirling pattern, and 4 defined as severe or intense echo density [16]. Table **1** displays the scoring systems.

Spontaneous echo contrast has been acknowledged to be present in the left ventricle among individuals with records of recent or remote anterior wall myocardial infarction and resulting wall motion abnormalities, as well as among dogs with experimentally induced anterior wall myocardial infarction [17]. The presence of spontaneous echo contrast in the left ventricle serves as an observable indication of abnormal contractility and a reduction in ejection fraction (EF). All of these conditions lead to a poor prognosis. Abnormal EF is usually followed by other comorbidities that affect survival [18]. Mahajan *et al*. demonstrated that the occurrence of LV-SEC is significantly higher in individuals with severe systolic dysfunction and sinus rhythm who have experienced cardioembolic stroke, compared to age- and gender-matched controls without stroke, with a statistically significant difference (*p* < 0.01). Nevertheless, the authors did not evaluate the potential autonomous influence of SEC on prognosis [19].

The prevalence of SEC detection is reported to be between 4.2% and 9.6% in patients with recent myocardial infarction [20]. In randomly selected populations undergoing transesophageal echocardiography (TEE), the frequency of SEC ranges from 3% to 20% [21, 22]. Bilge *et al*. have seen LA-SEC in 43% of hypertensive patients with LV systolic dysfunction and SR [23]. A large trial revealed that 21% of patients with stroke who were in sinus rhythm and had diminished function of the left ventricle had SEC on echocardiography [24]. Furthermore, the occurrence of SEC in individuals experiencing cerebral or peripheral embolic events ranges from 16% to 84% [25-27].

### 2.2 Correlation with Thromboembolic Events

The clinical significance of SEC resides in its correlation with embolic events. Intracavitary SEC not only signifies blood stagnation, which is a known precursor of thrombogenesis but also changes in blood characteristics, thereby reflecting two components of Virchow's triad of factors associated with thrombus formation [28]. Although a strong body of evidence supports that LA-SEC is associated with an elevated risk of thrombosis [7, 29-32], current data on the incidence of thromboembolic events in left ventricular spontaneous echo contrast are insufficient, with previous research being constrained by small sample sizes. Doud *et al*. [33] conducted a retrospective analysis of LV-SEC in 16 patients with cardiomyopathy, where they noted six thromboembolic events occurring in four patients. Another more recent investigation involving 98 cardiogenic shock patients receiving venoarterial extracorporeal membrane oxygenation (VA-ECMO) revealed a stroke prevalence of 36% in individuals with SEC (specifically 15 patients with LV-SEC) as detected by echocardiography [34]. In a study by Liang *et al*. [5], among 417 patients with LV-SEC, the incidence of 1-year embolism was 12.9%, a rate comparable to that observed in patients with left ventricular thrombus (LVT). Nevertheless, the inquiry persists over whether patients with LV-SEC, who exhibit thromboembolic events comparable to those with LVT, require anticoagulation management and what the appropriate dosage and duration would be for such treatment.

### 2.3 Anticoagulants in Left Ventricular Thrombus

LVT is a common condition that can lead to thromboembolism, which includes stroke, transitory ischemic attack, and extracranial systemic arterial embolism, such as limb ischemia [35, 36]. Studies have shown a correlation between LVT and systemic thromboembolism, with an incidence that can range anywhere from 3.7% to 16.3% [37-39]. From the year 2000 onwards, more than 4000 research studies on “left ventricular thrombus” have appeared in the PubMed database, emphasizing notable alterations in susceptible patient demographics, imaging modalities, and therapeutic alternatives. The scarcity of high-quality evidence from randomized trials has resulted in a dearth of guideline recommendations for managing this condition. However, recent years have witnessed considerable advancements in comprehending left ventricular thrombus and delineating strategies for its management. Consequently, the Scientific Statement provided by the American Heart Association, characterized by careful consideration and rationality, represents a pertinent and significant addition [40].

The statement provides eight practical management recommendations for patients who are at risk of or have LVT. Firstly, the benefit of oral anticoagulation (OAC) in preventing LVT in patients with anterior ST elevation myocardial infarction (MI) treated with reperfusion therapy is uncertain due to associated risks. Secondly, patients with LVT following MI should receive OAC for a duration of typically 3 months. Thirdly, prophylactic OAC should not be administered to patients with dilated cardiomyopathy, except in specific cases of higher-risk cardiomyopathies such as Takotsubo syndrome, LV non-compaction, and peripartum cardiomyopathy, where OAC may be considered. Fourthly, patients with non-ischemic cardiomyopathy and LVT should receive OAC for at least 3-6 months, with discontinuation if there is improvement in left ventricular ejection fraction (LVEF) or if bleeding occurs. Fifthly, newly diagnosed mural thrombus should be managed with oral anticoagulation in a manner analogous to protruding thrombus. Sixthly, Cardiac magnetic resonance (CMR) imaging is indicated when echocardiography suggests a thrombus without a definitive diagnosis or when echocardiography fails to detect a thrombus despite a high suspicion of thromboembolism. Seventhly, direct-acting oral anticoagulants (DOACs) can be considered a reasonable alternative to warfarin for the treatment of LVT. Lastly, if persistent LVT is present, particularly if it is protruding, it may be reasonable to try an alternative DOAC or low molecular-weight heparin. Additionally, it is reasonable to discontinue DOAC in cases where the thrombus undergoes organization or calcification.

The recommendations address the key inquiries raised by providers, each supported by a thorough review of the current literature. Most of the data originates from observational studies, which carry inherent limitations. Consequently, the recommendations are articulated with caution to recognize the uncertainty and the need for clinical judgment in evaluating risk and benefit. The duration of anticoagulation therapy for patients with dilated cardiomyopathy and a detected LVT remains unanswered due to insufficient study data. Clinical judgment may favor prolonged oral anticoagulation administration in cases of sizable and mobile LVT or when identified during an acute embolic stroke in patients with chronic dilated cardiomyopathy. In the context of an acute anterior myocardial infarction, especially with delayed presentation or absence of reperfusion therapy, both the imaging modality and the timing of the imaging procedure are critical factors.

On the choice of anticoagulation, observational studies, as summarized in this scientific statement, indicate that warfarin effectively reduces thromboembolic events in these patients [40]. Additionally, other observational studies have indicated similar rates of clinical events and thrombus resolution when comparing DOACs to warfarin. DOACs present appealing alternatives to warfarin due to their potential efficacy and safety; however, there is currently no randomized controlled trial demonstrating the effects of DOACs in LVT [41]. The increasing interest in DOACs stems from their straightforward administration, absence of INR monitoring or dietary limitations, and enhancement of overall quality of life [42]. The recommendations of guidelines on the treatment of LVT are shown in Table **2**.

Another interesting finding by Sonaglioni *et al*. [43] was the diagnostic and prognostic role of pulsed wave tissue Doppler imaging (PW-TDI) in patients with LVT. The objective was to assess if thrombus motion characteristics, specifically antegrade velocity (Va), could serve as predictors for major adverse cardiovascular events (MACE), especially embolic complications, within a one-year timeframe. A prospective study was conducted involving 72 patients with echocardiographically confirmed LVT, assessing velocity and other mobility parameters through pulsed-wave tissue Doppler imaging. Thrombi exhibiting high mobility (Va ≥10 cm/s) were significantly correlated with elevated embolic risk, demonstrating 94% sensitivity and 85% specificity in predicting major adverse cardiovascular events (MACE). The findings underscore the utility of PW-TDI as a bedside instrument for stratifying embolic risk and informing anticoagulant therapy in patients with mobile LV thrombi.

### 2.4 Anticoagulants in Patients with Heart Failure with Sinus Rhythm

The role of anticoagulation therapy in patients with chronic HF has been debated for many years. Anticoagulation has demonstrated efficacy in decreasing the incidence of thromboembolic events, especially strokes, in preliminary controlled clinical trials. However, these studies encompassed a diverse range of patients with HF, including those with atrial fibrillation (AF) and valvular heart disease, while featuring a comparatively small cohort of patients in SR [44-46]. The role of anticoagulants in patients with HFrEF and normal sinus rhythm is unclear, and there is significant variation in the utilization of anticoagulation in these patients. Important clinical trials aiming to determine the optimal therapy for minimizing thromboembolism and mortality risk in patients with left ventricular systolic dysfunction and SR include the WATCH [45] (Warfarin and Antiplatelet Therapy in Chronic Heart Failure) trial and the WARCEF [47] (Warfarin *versus* Aspirin in Reduced Cardiac Ejection Fraction) trial. The combined data from the WATCH and WARCEF studies may provide sufficient statistical power to clarify stroke and death outcomes in patients with a reduced cardiac EF. Additionally, the HELAS study concluded that thromboembolic events in patients with HFrEF in SR are infrequent, and there is currently no evidence to suggest that anticoagulant or antiplatelet treatment can reduce the occurrence of such events [48]. However, the COMMANDER-HF study reported the beneficial effects of anticoagulant therapy with rivaroxaban in patients with HFrEF in SR as the rate of stroke was dropped from 16.2 per 1000 patient-years in the group receiving placebo to 10.8 per 1000 patient-years in the group receiving rivaroxaban (HR 0.67, 95% CI 0.47–0.95) [49]. Additionally, Avellana *et al*. [50] conducted a study in order to evaluate the proper anticoagulation approach in heart failure patients who have sinus rhythm and do not have conditions such as AF, thrombus, or thromboembolic events in their medical records. Among a substantial group of patients diagnosed with HFrEF in SR, 26% received anticoagulation therapy. No correlation was found between this and reduced mortality or stroke incidence; however, a decrease in major cardiac events was observed. The uncertainty regarding the balance between the advantages and potential hazards of utilizing any antithrombotic medication in individuals with chronic heart failure in sinus rhythm persists [48].

A number of epidemiological studies have established an association between the time-dependent risk of stroke and heart failure in patients. Alberts *et al*. [51] identified a fourfold increase in stroke risk during the month following the diagnosis of HF within a population-based cohort (consisting of 7546 patients, 233 of whom had HF at baseline) monitored since 1990. The increased risk normalized within six months of follow-up in patients who survived. Prior research has indicated a heightened risk of stroke occurring shortly following the diagnosis of heart failure [52-55]. Witt *et al*. similarly reported a 17.4-fold increase in stroke risk within the first month of HF diagnosis, which decreased to a 2.9-fold increase after five years in a random sample of HF patients from Rochester who were followed for a longer duration [54]. Another study by Dries *et al*. [53] investigated the frequency and extent of thromboembolic events in patients with moderate to severe left ventricular systolic dysfunction and normal SR, as well as investigating the correlation between EF and the risk of thromboembolism. The incidence of thromboembolic events in patients exhibiting moderate to severe left ventricular dysfunction and SR is minimal. There is mounting evidence suggesting that the risk of stroke during the first 30 days after the onset of HF may nearly double the five-year risk of stroke in HF, with a lingering but smaller effect lasting up to six months [56]. This may also hold true for the immediate period following a stroke [57, 58]. These critical timeframes may potentially serve as relative indications for anticoagulation therapy in HF due to the higher stroke rates. However, further data are required to determine if anticoagulation would be justified in these situations by examining the incidence of stroke. Clinical trials on management of HF patients in SR are shown in Table **3**.

### 2.5 Management of Heart Failure Patients with LV-SEC and SR

Anticoagulation therapy carries inherent risks, necessitating careful consideration by clinicians in weighing the advantages of risk reduction against the potential for hemorrhage in patients undergoing anticoagulant therapy. Notably, guidelines currently lack sufficient data regarding the administration of anticoagulation in patients with HFrEF and sinus rhythm for both primary and secondary prevention of ischemic or embolic stroke [59]. In other words, there exists no conclusive evidence to support the use of antiplatelets or anticoagulation in all HF patients, even though HF is linked to hypercoagulability and increased platelet activation [60]. Treating patients with chronic heart failure using antithrombotic agents in the absence of a precise indication, such as recent coronary stenting, atrial fibrillation, implanted prosthetic valves, or ventricular assist devices, is a subject of controversy. Currently, it is not advisable to regularly administer these agents to such patients [59]. Hence, there is a lack of agreement regarding the necessity of administering prophylactic anticoagulation to these patients or the optimal timing for initiating such anticoagulant therapy.

The presence or absence of SEC/LVT could hold significant relevance in the therapeutic approach to HFrEF, even among patients with sinus rhythm. Due to the absence of major clinical trials, the efficacy of anticoagulation therapy aimed at stroke prevention was not firmly established in patients with HFrEF lacking atrial fibrillation or a history of thromboembolic events, thus limiting early guideline recommendations.

Various studies have indicated that the presence of SEC in the left atrium (LA-SEC) and left atrial appendage (LAA) is associated with an increased risk of systemic embolization [61-67]. Nevertheless, anticoagulant therapy has been administered to specific individuals diagnosed with HFrEF and LVT, yet its application hasn't been investigated in patients exhibiting SEC originating from the left ventricle. SEC is believed to induce a hypercoagulable condition, with its presence linked to coagulation indicators such as D-dimers, β-thromboglobulin, von Willebrand factor, and hematocrit [10, 68, 69]. SEC indicates blood stasis and is a known precursor of thrombus formation. The embolic risk in patients with LV-SEC is listed in Table **4**.

Zhou *et al*. [70] conducted a study aiming to ascertain the prevalence, predictive factors, and correlation with the risk of ischemic stroke concerning SEC or LVT among patients diagnosed with HFrEF. Retrospective analysis was conducted on clinical, echocardiographic, and follow-up data spanning from January 2009 to February 2019, obtained from medical records of individuals admitted with heart failure and LVEF < 40% as determined by echocardiography, with follow-up extended until February 2020. This study showed that among 9485 consecutive patients diagnosed with HFrEF, the prevalence of LVT and SEC was 1.3% and 3.5%, respectively. In multivariate analysis, SEC and LVT were found to correlate with male gender, age, smoking, ischemic cardiomyopathy (ICM), apical aneurysm, chronic kidney disease (CKD), LVEF, and left ventricular end-diastolic volume (LVEDV). In comparison to controls, the risk of stroke is two times higher in individuals with SEC and four times higher in those with LVT. In the absence of anticoagulant therapy, the presence of SEC was associated with a 2.5-fold increase in the risk of stroke embolism in patients with HFrEF. Conversely, patients with SEC/LVT receiving anticoagulation exhibited a risk of ischemic stroke events comparable to those without SEC/LVT. Additionally, patients in sinus rhythm with SEC/LVT and not receiving anticoagulant treatment faced a two-fold to five-fold increased risk of ischemic stroke.

In another study, Liang *et al*. [5] sought to elucidate the clinical attributes and outcomes of individuals with LV-SEC. This retrospective, single-center study enrolled patients who underwent consecutive echocardiograms from October 2009 to September 2019. Participants exhibiting LV-SEC were included, while those complicated by LVT, a history of infective endocarditis, prosthetic valves, or those lost to follow-up were excluded. The primary clinical endpoint was the occurrence of thromboembolic events (such as stroke and peripheral embolism) within one year. This study underscores that among the 417 patients (mean age 63.5 ± 14.7 years; 86.8% male), thromboembolic complications arise in 12.9% of patients with LV-SEC, prompting consideration of the potential benefits of anticoagulant therapy in this cohort. Age, AF, hemoglobin, and LVEF were independent risk factors for 1-year thromboembolic event in these patients.

Studies listed in Table **4** demonstrated greater thromboembolic risk in patients with LV-SEC in different settings. Recent studies have shown that anticoagulant use may improve the outcomes in patients with SEC/LVT and HFrEF, as anticoagulant therapy was found to be associated with reduced risk of thromboembolic events in participants with HF and LV-SEC with SR. Nonetheless, the ability to apply the findings of these studies to a wider population is restricted due to various factors. Selection biases, retrospective design, small sample size, duration, and dose of anticoagulant were not included in these analyses. Therefore, future investigations with a larger sample size are certainly required to verify the obtained results.

## CONCLUSION

In summary, anticoagulant therapy is recommended for most patients diagnosed with LVT by using vitamin K antagonists (VKA) or alternatives like DOACs for at least 3 to 6 months, which is the estimated duration required for thrombus resolution. In patients with heart failure and sinus rhythm, the risk of stroke is strongly increased in the early phase after the diagnosis, which could serve as a relative indication for anticoagulation therapy. Nevertheless, due to the attenuation of thromboembolic events over time, the benefits and risks of using any antithrombotic agent in patients with chronic heart failure in sinus rhythm remain uncertain. This review highlights that, in heart failure patients with sinus rhythm and spontaneous echo contrast (SEC) in the left ventricle, the presence of SEC is associated with poor left ventricular contraction and wall motion abnormalities. These conditions induce blood stasis, fulfilling one of Virchow’s triad factors that predispose to thrombogenesis. Additionally, the reviewed studies revealed that patients with LV-SEC have a higher incidence of thromboembolism despite sinus rhythm, and anticoagulant therapy may improve their outcomes. Nonetheless, future studies are still required to establish the appropriate management for these patients, including the validity, duration, and dosage of anticoagulation therapy.

## Figures and Tables

**Table 1:** Grading systems for SEC (mostly LA-SEC).

**Score**	**Interpretation**
**Sadanandan *et al*. [** **14** **]**	
Score	Interpretation
0	No smoke
+1	Mild smoke is visible in some portions of LA
+2	Dense smoke that appeared throughout the LA
**Watanabe *et al*. [** **15** **]**	
0	No SEC
+1	Mild, indicating minimal echogenicity in the left atrial appendage
+2	Moderate, and demonstrated a dense swirling pattern in the main cavity
+3	Severe and demonstrated intense echodensity, with very slow swirling patterns in the main cavity
**Fatkin *et al*. [** **16** **]**	
0	None (absence of echogenicity)
+1	Mild (minimal echogenicity located in the LA appendage or sparely distributed in the main cavity of the left atrium; may be detectable only transiently during the cardiac cycle; imperceptible at operating gain settings for two dimensional echocardiographic analysis)
+2	Mild to moderate (more dense swirling pattern than grade 1+ but with similar distribution; detectable without increased gain settings)
+3	Moderate (dense swirling pattern in the left atrial appendage; generally associated with somewhat lesser intensity in the main cavity; may fluctuate in intensity but detectable constantly throughout the cardiac cycle)
+4	Severe (intense echo density and very slow swirling patterns in the LAA, usually with similar density in the main cavity)

**Abbreviations:** SEC, spontaneous echo contrast; LA, left atrium; LA-SEC, left atrial spontaneous echo contrast, LAA, left atrial appendage.

**Table 2:** Guidelines on treatment of left ventricular thrombosis.

**Guideline / Year**	**Recommendation**	**Class of Recommendation/** **Level of Evidence (LOE)**
ACCP [71] /2012	1) For LVT or high-risk anterior MI without stenting, use warfarin (INR 2-3) plus low-dose aspirin for 3 months, then switch to DAPT for up to 12 months. 2) For LVT or high-risk anterior MI with BMS, use warfarin (INR 2-3), low-dose aspirin, and clopidogrel for 1 month; then warfarin plus SAPT for months 2–3; then switch to DAPT for up to 12 months per ACS guidelines. 3) For DES patients with LVT or high-risk anterior MI, use warfarin (INR 2-3) with low-dose aspirin and clopidogrel for 3–6 months, then switch to DAPT for up to 12 months per ACS guidelines. 4) For patients with LVT + systolic LV dysfunction without established CAD Warfarin (INR 2-3) for at least 3 months should be administered. 5) For High-risk patients for LVT without established CAD, no antiplatelet therapy or warfarin is required.	1) Grade 1B 2) Grade 2C 3) Grade 2C 4) Grade 2C 5) Grade 2C
ACC/AHA STEMI [72] /2013	1) VKA anticoagulation is advised for patients with asymptomatic mural thrombi. 2) Anticoagulation should be considered in patients exhibiting anteroapical dyskinesis on echocardiogram.	1) Class IIa, LOE C 2) Class IIb, LOE C
ESC STEMI [73] /2018	In the presence of a confirmed LVT, anticoagulation should be administered for a duration of up to six months, with management informed by serial imaging assessments.	Class IIa, LOE C
AHA/ASA STROKE [74] /2021	1) Patients with stroke or TIA and LV thrombus should receive anticoagulation with therapeutic warfarin for a minimum of 3 months to mitigate the risk of recurrent stroke. 2) In patients experiencing stroke or TIA concurrent with acute myocardial infarction, it is appropriate to conduct advanced cardiac imaging (*e.g.*, contrasted echocardiogram or cardiac MRI) to evaluate for LVT presence. 3) The safety of anticoagulation using a direct oral anticoagulant to mitigate the risk of recurrent stroke in patients with stroke or TIA and new LVT (within 3 months) remains uncertain. 4) In patients experiencing stroke or TIA during anterior STEMI with EF <50% and no evidence of LVT, empirical anticoagulation for a minimum of 3 months may be warranted to mitigate the risk of recurrent cardioembolic stroke.	1) Class 1/B-NR 2) Class 2a/C-EO 3) Class 2b/C-LD 4) Class 2b/C-EO

**Abbreviations:** ACCP, American college of chest physicians; ACC/AHA, American College of Cardiology /American Heart Association; AHA/ASA, American Heart Association/American Stroke Association; ESC, European Society of Cardiology; STEMI, ST-elevation myocardial infarction; LVT, left ventricular thrombus; MI, myocardial infarction; INR, international normalized ratio; DAPT, dual antiplatelet therapy; ACS, acute coronary syndrome; BMS, bare metal stent; DES, drug-eluting stent; SAPT, Single-antiplatelet therapy; CAD, coronary artery disease; VKA, vitamin K antagonist; TIA, transitory ischemic attack; MRI, magnetic resonance imaging.

**Table 3:** Clinical trials on anticoagulant therapy in heart failure patients in sinus rhythm.

**Reference / Year**	**Design**	**Participant**	**Conclusion**
The WASH study [75] / 2004	Randomized Controlled trial	279 M/W >60 years	The WASH study found that antithrombotic therapy in heart failure patients is unsupported and often leads to polypharmacy. Warfarin may not work for sinus rhythm heart failure.
The HELAS study [49] / 2006	Randomized Controlled trial	197 M/W >40 years	In heart failure, regardless of the treatment, the occurrence of embolic events is generally infrequent. The efficacy of treatment does not appear to have an impact on the final result.
The WARCEF trial [48] / 2006	Randomized Controlled trial	2860 M/W >18 years	The WARCEF trial demonstrated no significant difference in outcomes between warfarin (INR 2.0-3.5) and aspirin (325 mg/day) over a follow-up period of 3 to 5 years.
The WATCH trial [76] / 2009	Randomized Controlled trial	1587 M/W >50 years	The Program ended the trial 18 months early due to low enrollment. The primary outcome measure and mortality data do not support the hypotheses that warfarin and clopidogrel are better than aspirin.
Warfarin and aspirin in patients with heart failure and sinus rhythm [58] / 2012	Randomized Controlled Trial	2305 M/W >50 years	The primary outcome was not significantly different between warfarin and aspirin in sinus rhythm patients with reduced LVEF. The choice between warfarin and aspirin must be individualized for each patient.
Analysis of REDINSCOR registry [51] / 2012	Retrospective observational study	2263 M/W >50 years	There was no correlation between undergoing anticoagulation therapy and a decrease in death rate or occurrence of strokes.
Rivaroxaban in patients with heart failure, sinus rhythm, and coronary disease [59] / 2018	Randomized Controlled Trial	5022 M/W >55 years	Rivaroxaban at 2.5 mg twice daily did not reduce mortality or cardiac events compared to a placebo in HFrEF patients with coronary artery disease and no AF.
The COMMANDER HF trial [50] / 2019	Randomized Controlled trial	5022 M/W >50 years	Rivaroxaban 2.5 mg b.i.d. reduced stroke and TIA risk in patients with HFrEF and CAD.

**Abbreviations:** M, man; W, woman; WASH, Warfarin/Aspirin Study in Heart failure; WATCH, Warfarin and Antiplatelet Therapy in Heart Failure Trial; HELAS, Heart failure Long-term Antithrombotic Study; WARCEF, Warfarin *vs*. Aspirin in Reduced Cardiac Ejection Fraction; HFrEF, heart failure with reduced ejection fraction; AF, atrial fibrillation; TIA, transitory ischemic attack; CAD, coronary artery disease; b.i.d, every 12 hours; LVEF, left ventricle ejection fraction.

**Table 4:** Embolic risk in patients with LV-SEC.

**Method**	**Result**	**References / Year**
Sixteen patients with LV-SEC on two-dimensional echocardiograms were retrospectively analyzed for clinical characteristics, reproducibility, thrombi and embolic events, and prognostic implications. The average EF was 17.6%.	Four patients experienced six thromboembolic events; spontaneous contrast persisted up to 39 months and disappeared with improved LV function.	Doud *et al*. [33] / 1990
Retrospective study of 89 spontaneous echo contrast patients (67% male, 53.9 ± 14.3 years) found ischemic heart disease in 68%, non-ischemic cardiomyopathy in 19.3%, rheumatic valvular disease in 12.5%, with hypertension (54%), renal insufficiency (34%), diabetes (34%), and atrial fibrillation (19%); 90% had left ventricular SEC, mean EF was 34.8 ± 16.3% (64% <35%), and most showed segmental/global hypokinesia.	The occurrence of cardio-embolic events was 10%, with 9.0% manifesting as stroke and 1.0% presenting as acute limb ischemia.	Nelson *et al*. [77] / 2014
Ninety-eight cardiogenic shock patients who were undergoing venoarterial extracorporeal membrane oxygenation (VA-ECMO) were studied. Patients were divided into 2 groups based on the presence or absence of LV-SEC on echocardiography.	The LV-SEC group had higher rates of intracardiac thrombus (46% *vs*. 13%) and stroke (36% *vs* 7.9%). Univariate analysis identified intracardiac thrombus, LV-SEC, and low pulsatility as stroke risk factors. In multivariate analysis, LV-SEC was the only independent stroke risk factor.	Unai *et al*. [34] / 2017
Data from 9,485 HFrEF patients (LVEF <40%) collected from January 2009 to February 2019 (follow-up until February 2020) showed 123 (1.3%) had LVT and 331 (3.5%) had LV-SEC.	SEC and LVT served as independent predictors for the occurrence of ischemic stroke. In patients with HFrEF, SEC has a prevalence of 1.3% and associated with an elevated risk of ischemic stroke and LVT.	Zhou *et al*. [71] / 2021
A retrospective study (Oct 2009–Sept 2019) included LV-SEC patients, excluding those with LVT, infective endocarditis, prosthetic valves, or lost follow-up, to assess one-year thromboembolic events.	In a cohort of 417 patients (mean age 63.5 ± 14.7 years; 86.8% male) diagnosed with LV-SEC, the 1-year incidence of embolism was found to be 12.9%.	Liang *et al.* [5] / 2021

**Abbreviations:** SEC, spontaneous echo contrast; LV-SEC, left ventricular spontaneous echo contrast; EF, ejection fraction; LVEF, left ventricular ejection fraction; HFrEF, heart failure with reduced ejection fraction; LVT, left ventricular thrombus.

## LIST OF ABBREVIATIONS

HF: Heart Failure
LV-SEC: Left Ventricular Spontaneous Echo Contrast
LVT: Left Ventricular Thrombus
MI: Myocardial Infarction
OAC: Oral Anticoagulation
SEC: Spontaneous Echo Contrast
SR: Sinus Rhythm
